# Controlled Impact Fragmentation of Extracted Teeth as a Novel Strategy for Dentin Graft Preparation: A Granulometric and Comparative Study

**DOI:** 10.3390/ma19194176

**Published:** 2026-09-30

**Authors:** Pablo Moreno Garibaldi, Tannia Calderon Avila, Rafael Carrera Espinoza, Melvyn Alvarez Vera, Juan Alfonso Beltrán Fernández, María Teresa Jiménez Munguía, Adriana Palacios Rosas, Christian Lagarza Cortes

**Affiliations:** 1 Departamento de Ingeniería Industrial y Mecánica, Escuela de Ingeniería, Universidad de las Américas Puebla, Ex-Hacienda Santa Catarina Mártir S/N, Puebla 72810, Mexico; tannia.calderonaa@udlap.mx (T.C.A.); rafael.carrera@udlap.mx (R.C.E.); melvyn.alvarez@udlap.mx (M.A.V.); adriana.palacios@udlap.mx (A.P.R.); christian.lagarza@udlap.mx (C.L.C.); 2 Instituto Politécnico Nacional Escuela Superior de Ingeniería Mecánica y Eléctrica, Sección de Estudios de Posgrado e Investigación Edificio 5, 2do Piso, Unidad Profesional Adolfo López Mateos, “Zacatenco” Col. Lindavista, Ciudad de Mexico 07738, Mexico; jbeltranf@hotmail.com; 3 Departamento de Ingeniería Química, Alimentos y Ambiental, Escuela de Ingeniería, Universidad de las Américas Puebla, Ex-Hacienda Santa Catarina Mártir S/N, Puebla 72810, Mexico; mariat.jimenez@udlap.mx

**Keywords:** xenografts, particle size, dentin, bone regeneration, dental implants, bone transplantation

## Abstract

Alveolar bone preservation following tooth extraction requires biomaterials with appropriate biological and physical properties. Here, particle size and morphology of the biomaterial play a critical role in bone regeneration. Tooth particle grafts represent a promising alternative because of their compositional similarity to bone; however, current dentin processing methods often lack standardization and produce heterogeneous particle populations. This study evaluated a novel impact-driven fragmentation system designed to produce dentin particles with controlled size distributions through adjustment of a predefined clearance distance. Fresh porcine teeth were fragmented under three operating conditions (250, 300, and 400 µm clearance distances). Particle characterization was performed using scanning electron microscopy (SEM), laser diffraction granulometric analysis, morphometric measurements of particle length and width, descriptive statistical analysis, and linear regression analysis to evaluate dimensional relationships. Laser diffraction analysis demonstrated that increasing clearance distance produced progressively larger particles, with volume mean diameters of 222, 289.6, and 356 µm for the 250, 300, and 400 µm conditions, respectively. The 300 µm condition generated the most homogeneous particle population, exhibiting the lowest span value (0.34) and the narrowest particle size distribution. SEM observations revealed elongated particles with consistent morphology across all conditions. Morphometric analysis confirmed proportional increases in particle length and width with increasing clearance distance, while aspect ratios remained relatively constant (2.02–2.37). Regression analysis showed a positive linear relationship between particle length and width, supporting a common fragmentation mechanism across all operating conditions. Controlled impact fragmentation enables predictable dentin particle production, allowing for particle size regulation through a defined mechanical parameter while preserving particle morphology. The 300 µm clearance condition provided the most favorable balance between particle size control and distribution homogeneity, suggesting that this approach may contribute to the standardized preparation of tooth-derived graft biomaterials for future bone regeneration applications.

## 1. Introduction

Tooth extraction initiates a series of biological processes driven by the inflammatory response resulting from the surgical procedure, as well as the absence of masticatory stimulation in the periodontium, which compromises the structural integrity of the periodontal tissues. As a result, a physiological phenomenon known as disuse atrophy occurs, characterized by significant alveolar bone resorption during the first weeks following the extraction [1]. These alterations begin with the resorption of bone that borders the extraction socket, leading to a vertical and horizontal bone volume reduction on the manipulated surface [1,2,3]. This reduction can take up to 50% of the ridge width during the first year following the extraction, with approximately two-thirds of the total alterations taking place within the first three months post-extraction, corresponding to a loss of approximately 5–7 mm [4,5]. The dimensional reduction in the alveolar ridge following tooth extraction not only changes the structural integrity of the periodontal tissue; it also jeopardizes the functional and aesthetic restoration of the edentulous area. The gradual loss of ridge height and width can impede the proper placement and stability of future prosthetic restorations, particularly dental implants, which require an adequate quantity and quality of alveolar bone for successful osseointegration and long-term maintenance. Retaining the post-extraction ridge dimensions is seen as a critical step in assuring optimal implant placement circumstances and predictable functional and aesthetic outcomes [6].

Numerous efforts in the search for suitable materials to preserve alveolar bone have led to the development of various grafting techniques and biomaterials. A biomaterial intended for bone augmentation must satisfy certain characteristics, such as biocompatibility, which refers to the capability of providing osseointegration without causing inflammatory and allergic reactions over time [7,8]; osteoconductivity, which allows for cell activity, reproduction, and amplification, and provides structure for the apposition of new bone [9,10]; and osteoinductivity, which refers to the capability of inducing biochemical and regulatory processes, so stem cells can differentiate into osteoblasts, osteoclasts, and osteocytes and induce osteogenesis, which is associated with the formation of a new bone matrix [11,12,13]. Thus, the concept of autogenous bone graft emerges, regarded as “the gold-standard biomaterial in bone regeneration procedures” as a result of its osteogenic, osteoinductive, and osteoconductive properties, which are biologically plausible given the compositional similarity between tooth and bone tissues [3]. This is further evidenced by the observation that dentin’s composition is nearly identical to human bone in terms of organized calcium and phosphate ions, such as hydroxyapatite and tricalcium phosphate. Its organic matrix is rich in type I collagen and growth factors, while its microtubular structure facilitates bone formation and enhances osteoconduction, resulting in a performance superior to that of xenogeneic or other graft types [14,15]. In addition, it is important to highlight that the dental matrix has a porous microstructure that facilitates cell adhesion, blood circulation, and slow resorption, ensuring optimal osteoconduction and preserving bone volume for a longer period of time [16]. Furthermore, other studies demonstrate that dentin can induce new bone formation and gradually replace bone tissue without triggering inflammatory reactions. Its low immunogenicity and rapid healing capacity represent unique advantages not comparable to those of any other graft material [17].

When the dental matrix is appropriately processed, it retains osteoinductive properties associated with bone morphogenetic proteins (BMPs), which are naturally present in the teeth in physiological quantities [18,19]. Beyond composition, the grain size and physical characteristics of the graft material play a decisive role in the biological response, directly influencing resorption rate and cellular activity [20,21]. Although there is no universally established optimal grain size for bone graft materials, several studies report values ranging between 300 and 1200 µm [22,23,24]. Experimental evidence indicates smaller particles promote faster conversion to bone than larger ones, due to their increased surface reactivity [25].

Beyond particle size, the process used to obtain tooth-derived graft particles also plays a decisive role in clinical success. Tooth-based grafts require that extracted teeth undergo a controlled grinding procedure to generate particles that are functionally suitable for grafting applications. However, the literature shows substantial variability in how this processing is performed. There is no standardization in the treatment steps, composition, procurement procedures, or particle size dimensions [26]. This lack of standardization directly affects the physical and biological properties of the resulting biomaterial and ultimately undermines its predictability and clinical utility. A further source of variation lies in differences in the type of device used, the handling of the processed dentin, and the inclusion or exclusion of enamel [3]. For example, a study generated by Dlucik, in which three different dentin-processing methods were compared, demonstrated noticeable differences in the characteristics of the particles obtained. Dentin matrices prepared using automated grinding devices, such as the Smart Dentin Grinder Genesis [22] and the BonMaker, showed distinct particle morphologies associated with a predominantly mechanical grinding and sieving process, resulting in irregularly shaped granules with heterogeneous surface features. At higher magnification, these reliefs exhibited a lamellar shape, featuring dentinal tubules and orifices. Some particles displayed deeper and orderly rows, especially on their lateral surfaces. In contrast, dentin processed with the Tooth Transformer exhibited a different structural organization, attributed to the combined mechanical, chemical, and thermal treatment applied during preparation, which led to modifications in the surface structure of the dentin particles. Scanning electron microscopy revealed qualitative differences in surface texture and microstructural appearance among the three biomaterials; however, despite these morphological variations, all particle types were considered suitable for clinical application [27,28].

To provide an alternative approach for dentin processing, in the present research, a novel method is proposed, with particular emphasis on the analysis of granulometry and particle size distribution, as these parameters are critical for achieving the biological and mechanical properties required for effective tooth-derived graft materials. The device operates through an impact-based mechanism in which rotational motion is transformed into controlled linear displacement. This conversion enables a mass to repeatedly enter the processing chamber and strike the tooth structure, progressively fragmenting it into smaller particles until a predefined distance and size range are achieved. Through this automated impact system, the device aims to generate biomaterial that is functionally appropriate for grafting applications. The resulting particles are subsequently analyzed and compared with those produced by other established processing methods.

## 2. Materials and Methods

### 2.1. Tooth Sample Collection and Pre-Processing

Fresh porcine teeth were obtained from a local slaughterhouse immediately after routine animal processing. Porcine teeth were selected as a xenogeneic source to evaluate the feasibility of producing a tooth-derived xenograft biomaterial, because of the similarities with human dentin, such as structural, compositional, and mineral [29,30].

After teeth collection, adherent soft tissues, periodontal ligament remnants, and surface debris were carefully removed using sterile manual instruments to avoid contamination and ensure consistent results. Teeth presenting extensive fractures or severe structural compromise were excluded from the study to minimize variability during impact-driven processing.

The cleaned specimens were thoroughly rinsed with distilled water and temporarily stored in a 0.1% thymol solution for disinfection and prevention of microbial proliferation while preserving the physicochemical integrity of the dentin matrix. Before fragmentation, the teeth were rinsed again with distilled water and air-dried at room temperature.

No chemical demineralization, thermal treatment, or enamel removal was performed before mechanical processing. This decision was made to evaluate exclusively the performance of the proposed impact-driven system in generating dentin particles intended for xenograft applications, and to assess its direct influence on particle size distribution and morphological characteristics.

### 2.2. Impact-Driven Fragmentation Device and Working Principle

**Figure 1:**
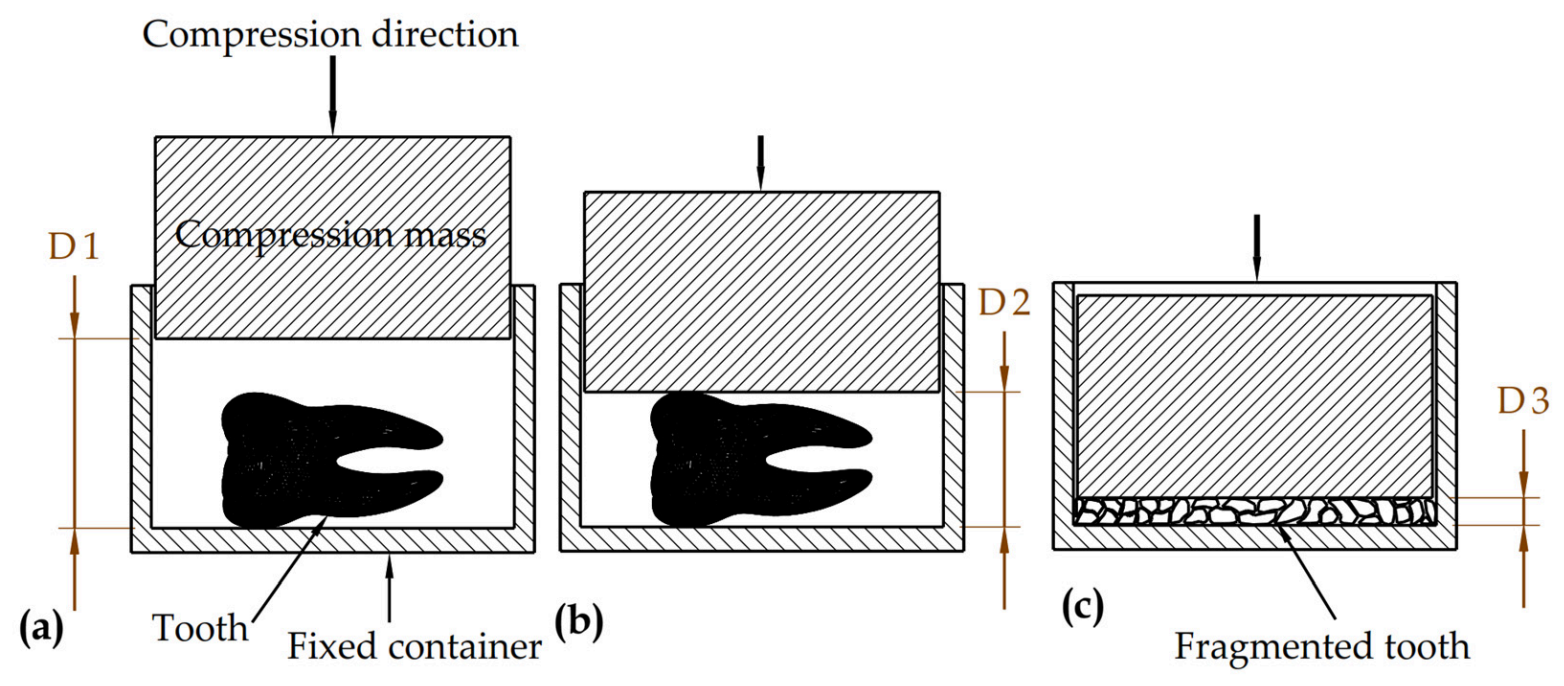
Impact-based device for tooth fragmentation working principle: (**a**) tooth prepared for fragmentation, (**b**) first contact compression mass-tooth, and (**c**) fragmented tooth with controlled distance.

A novel impact-based device was designed to fragment extracted teeth into graft-suitable particles. The system operates by converting rotational motion into controlled linear displacement, allowing a mass to move vertically within a confined chamber and repeatedly strike the tooth positioned inside a rigid container. A schematic representation of the progressive fragmentation mechanism is shown in Figure 1, illustrating the working principle of the linear displacement and the gradual reduction in particle size.

Figure 1 shows how the tooth specimen was placed within a cylindrical processing chamber fabricated from stainless steel to withstand repeated mechanical loading. A mobile impact mass of 0.52 kg was driven by a motorized mechanism that transforms angular motion into vertical displacement at 240 Hz. During operation, the impact mass enters the fixed container and progressively hits the tooth, generating fragmentation through repeated controlled impacts.

To control the final particle size, the impact-based system incorporates a mechanical blocking element that limits the minimum travel distance of the impact mass inside the processing chamber. By modifying the position of this stop element, predefined clearance distances of 250, 300, and 400 µm were established. Once the impact mass reaches the selected mechanical limit, further reduction in particle size is restricted, allowing for indirect control of the resulting particle size distribution through a repeatable geometric parameter. This approach enables particle size regulation during fragmentation rather than through subsequent classification or sieving operations.

### 2.3. Controlled Fragmentation Distances

To evaluate the influence of the predefined clearance distance on particle size distribution, three controlled fragmentation conditions were established: 600 µm, 450 µm, and 300 µm.

For each experimental condition, teeth were fragmented independently, ensuring that the fragmentation process was halted once the system reached the predefined distance. The objective was to obtain a particle population predominantly corresponding to the selected clearance while minimizing excessive over-grinding.

Each group was processed under identical operational parameters (impact frequency and mass weight), with the clearance distance being the only controlled variable. The resulting particles were collected, labeled according to their respective fragmentation distance, and stored in sterile containers before morphological and granulometric analysis.

### 2.4. Scanning Electron Microscopy (SEM) Analysis

The morphology of the samples was evaluated using a Tescan MAIA-3 field-emission Scanning Electron Microscope (TES-CAN, Brno, Czech Republic). The microscope was operated at an accelerating voltage of 10 kV and a working distance of 10.91 mm. High-vacuum imaging conditions were: gun pressure of <4.0 × 10^−8^ Pa, column pressure of 4.8 × 10^−3^ Pa, and chamber pressure of <1.0 × 10^−2^ Pa. These parameters correspond to the high-vacuum operating regime.

Random samples from each of the three experimental groups (600 µm, 450 µm, and 300 µm) were mounted on aluminum stubs using conductive carbon tape. SEM micrographs were obtained at different magnifications to assess particle shape and angularity.

Qualitative comparisons among the three fragmentation distances were performed to determine the morphology generated by the impact-driven method.

### 2.5. Particle Size Distribution by Laser Diffraction

**Figure 2:**
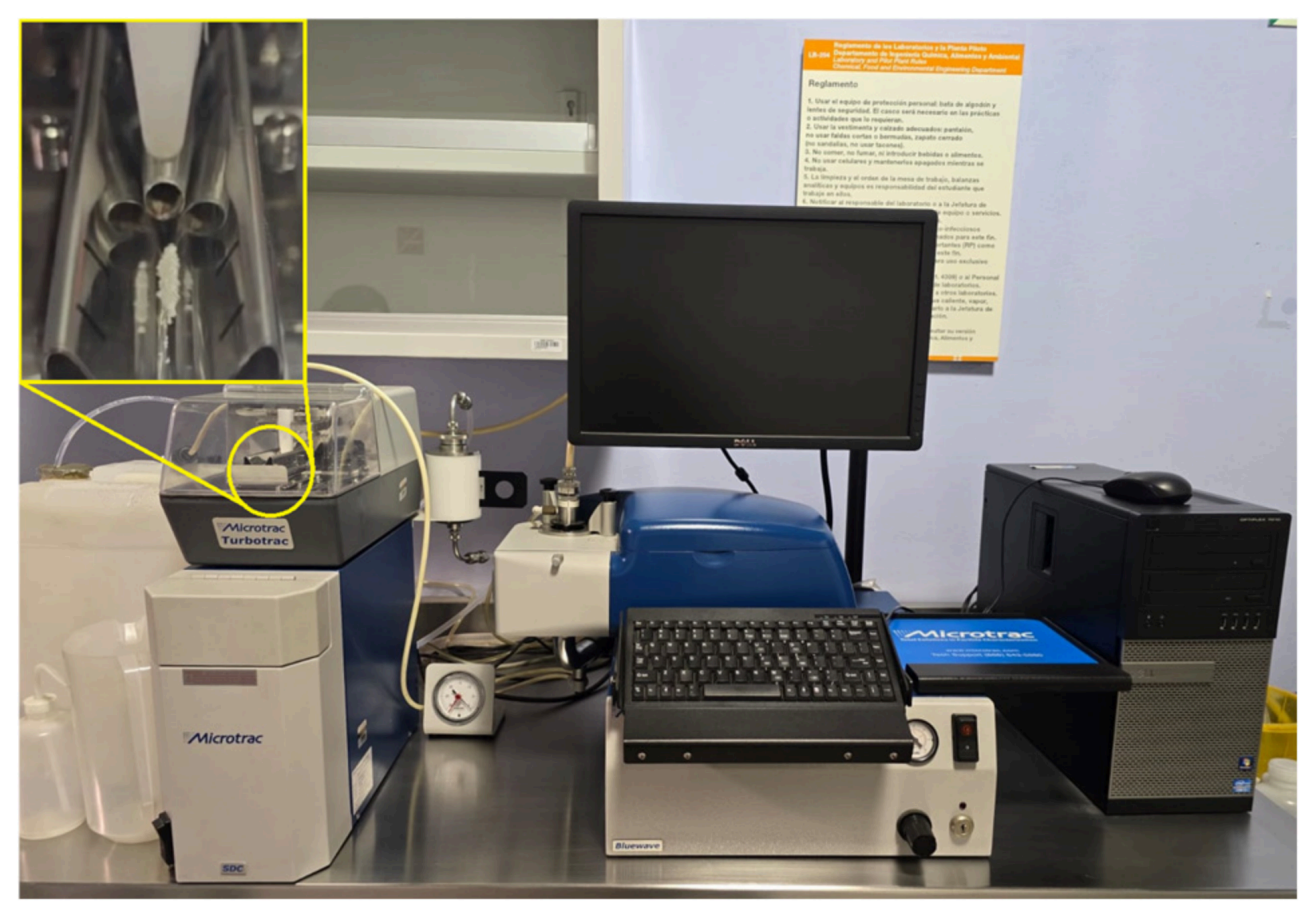
MICROTRAC S3500 used for granulometric characterization.

Granulometric characterization of the fragmented dentin particles was conducted using laser diffraction analysis with a MICROTRAC S3500 (Microtrack Inc., PA, USA), (Bluewave model) (see Figure 2).

Figure 2 shows how the sample was dispersed on the V-shaped container to prevent particle agglomeration before analysis. The system provided volumetric particle size distribution data, including:

- D10 (10% cumulative volume diameter);
- D50 (median particle diameter);
- D90 (90% cumulative volume diameter);
- Differential volume distribution.

Three independent measurements were performed for each fragmentation distance to ensure reproducibility. The obtained distributions were analyzed to determine the effectiveness of the impact-driven method in achieving targeted particle size ranges.

### 2.6. Morphometric Measurements (Length and Width)

In addition to laser diffraction analysis, direct morphometric measurements were performed on Scanned Electron Microscopy images to determine particle length and width.

For each fragmentation condition, at least 30 particles were randomly selected and measured. Mean values and standard deviations were calculated for both length and width. These measurements complemented the volumetric data obtained by laser diffraction and provided additional insight into particle geometry and aspect ratio.

### 2.7. Statistical Analysis

Descriptive statistics were used to summarize the granulometric and morphometric data. Results are reported as mean ± standard deviation. Regression analysis was performed to evaluate the relationship between particle length and width. Because the primary objective of the study was the characterization of particle populations generated under different operating conditions, the analysis focused on descriptive comparisons of the resulting distributions and morphological parameters.

## 3. Results

**Figure 3:**
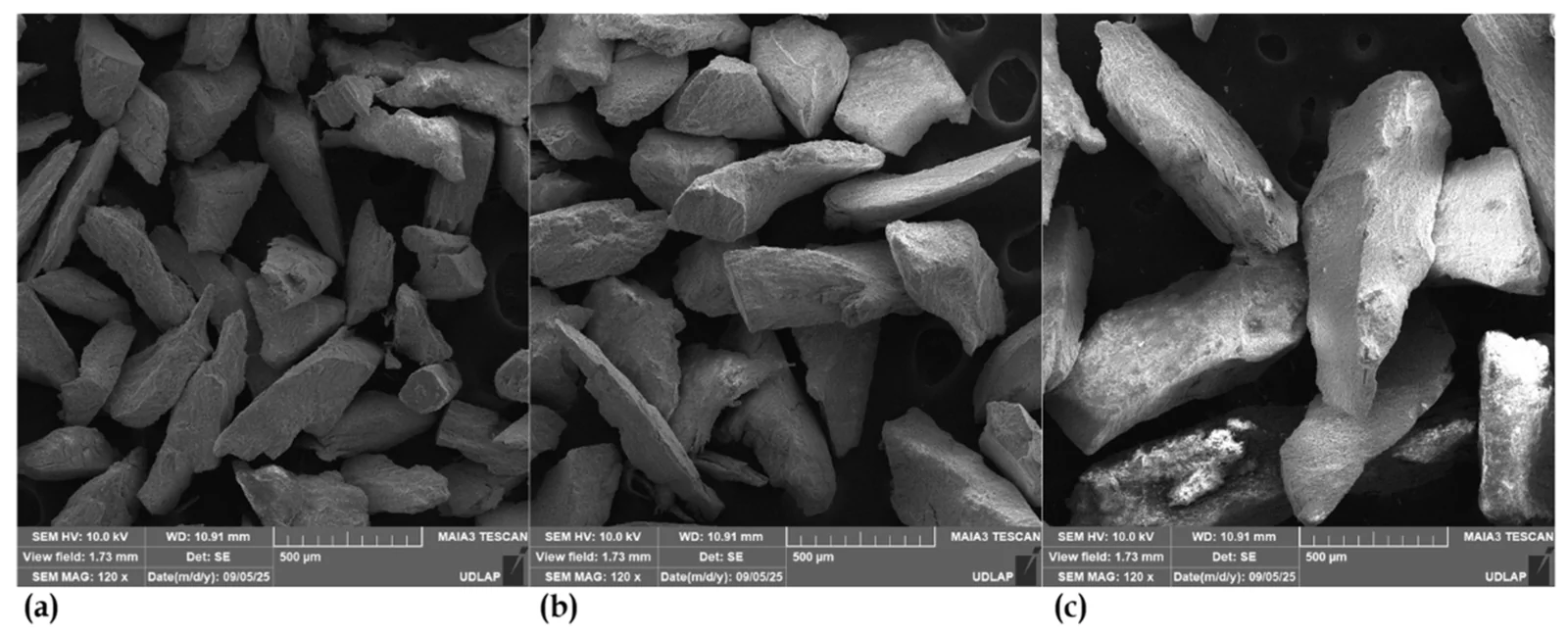
SEM images of the morphology of fragmented dentin particles at different clearance distances: (**a**) 250 µm, (**b**) 300 µm and (**c**) 400 µm.

These results provide an overview of the particle characteristics achieved under different clearance distances with controlled fragmentation conditions; the initial findings include qualitative observations from SEM, as is shown in Figure 3.

In Figure 3, for the three different clearance distances analyzed (a, b, and c), the morphology of particles appears relatively homogeneous, characterized by a narrow width and greater length, describing an elongated particle shape. This suggests that the fragmentation mechanism produces particles with similar geometric characteristics regardless of the clearance distance, indicating morphological consistency in the grinding process. Additionally, according to the dimensional characteristics of each group, they maintain a relatively uniform size, indicating a direct relationship between clearance distance and particle size.

**Figure 4:**
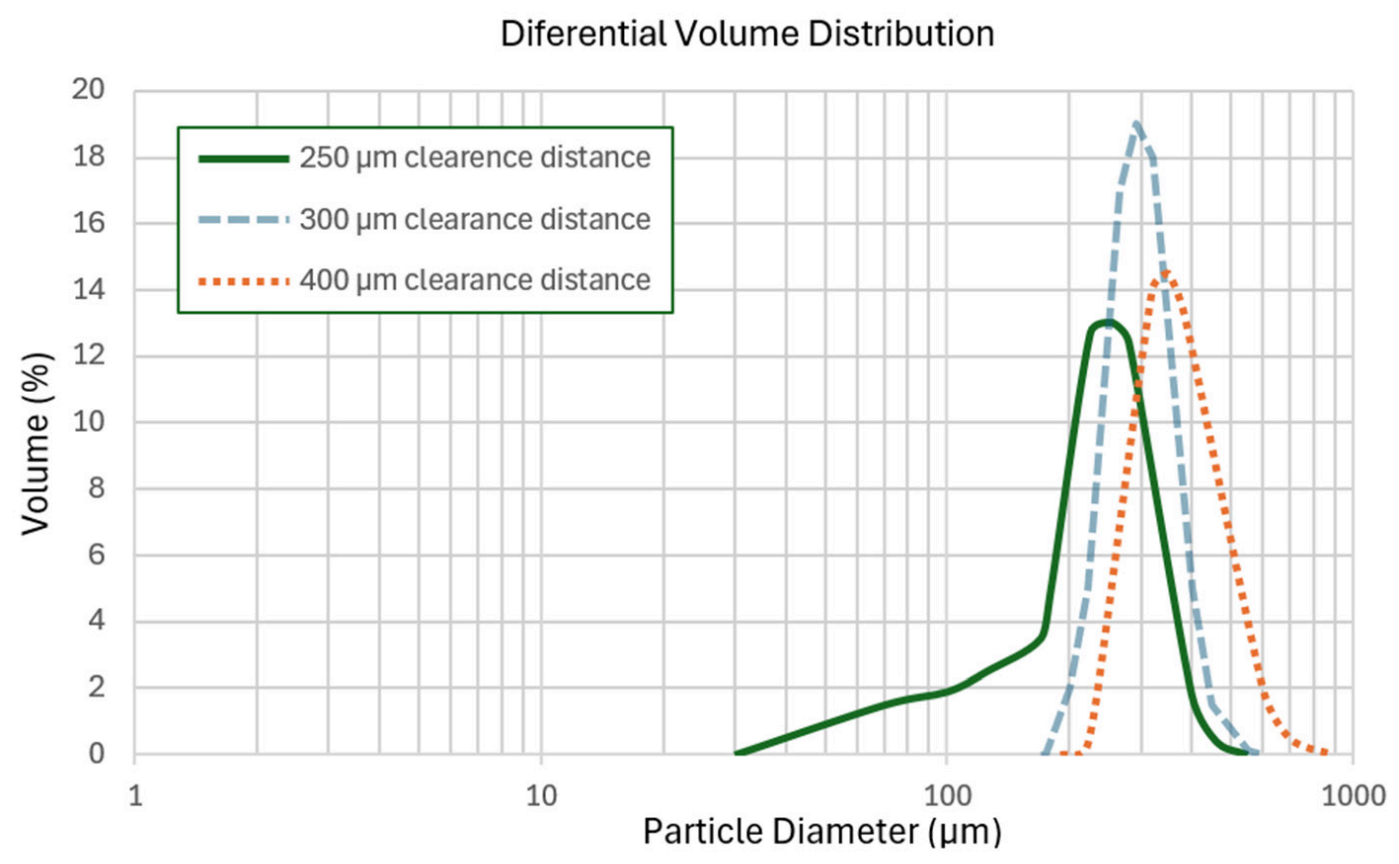
Differential Volume Distribution of particles at different clearance distances.

The results of particle size distribution using laser diffraction analysis for the three different samples are shown in Figure 4.

According to Figure 4, for the 300 µm clearance distance sample, the central tendency of the distribution is well within the intended experimental range of 200–300 µm, and it is very close to the volume mean ($M_{\mathrm{volume}}$ = 289.6 µm). In addition, according to Table 1, this sample presents a value of $Dv_{50}$ = 285.8 µm, and exhibits a remarkably narrow volume peak curve, where approximately 80% of the sample volume is concentrated in a range of less than 100 µm; this is supported by the difference of $Dv_{10}=\;$244.9 µm and $Dv_{90}=$ 340.8 µm. Additionally, a 300 µm clearance distance yielded the lowest span value (0.34), compared with 0.70 and 0.57 for the 250 µm and 400 µm conditions, respectively.

In the case of the other two samples, the results are similar. For the 400 µm sample, the volume mean ($M_{\mathrm{volume}}$ = 356 µm), and $Dv_{50}$ = 340.8 µm, having the 80% of the sample concentrated in less than 200 µm, taking as a reference $Dv_{10}=\;$271.5 µm and $Dv_{90}=$ 465.7 µm; and for the case of a 250 µm sample, the volume mean ($M_{\mathrm{volume}}$ = 222 µm), and $Dv_{50}$ = 227.1 µm, having the 80% of the sample concentrated in less than 160 µm, taking as a reference $Dv_{10}=\;$135.2 µm and $Dv_{90}=$ 293.5 µm, despite the presence of some finer particles below 150 µm. This outcome is expected when grinding complex biological materials such as teeth, particularly due to the brittle enamel and the more elastic dentin matrix that the teeth have, causing an unpredictable response to mechanical impact stress, generating a minor population of sub-150 µm fine particles.

**Figure 5:**
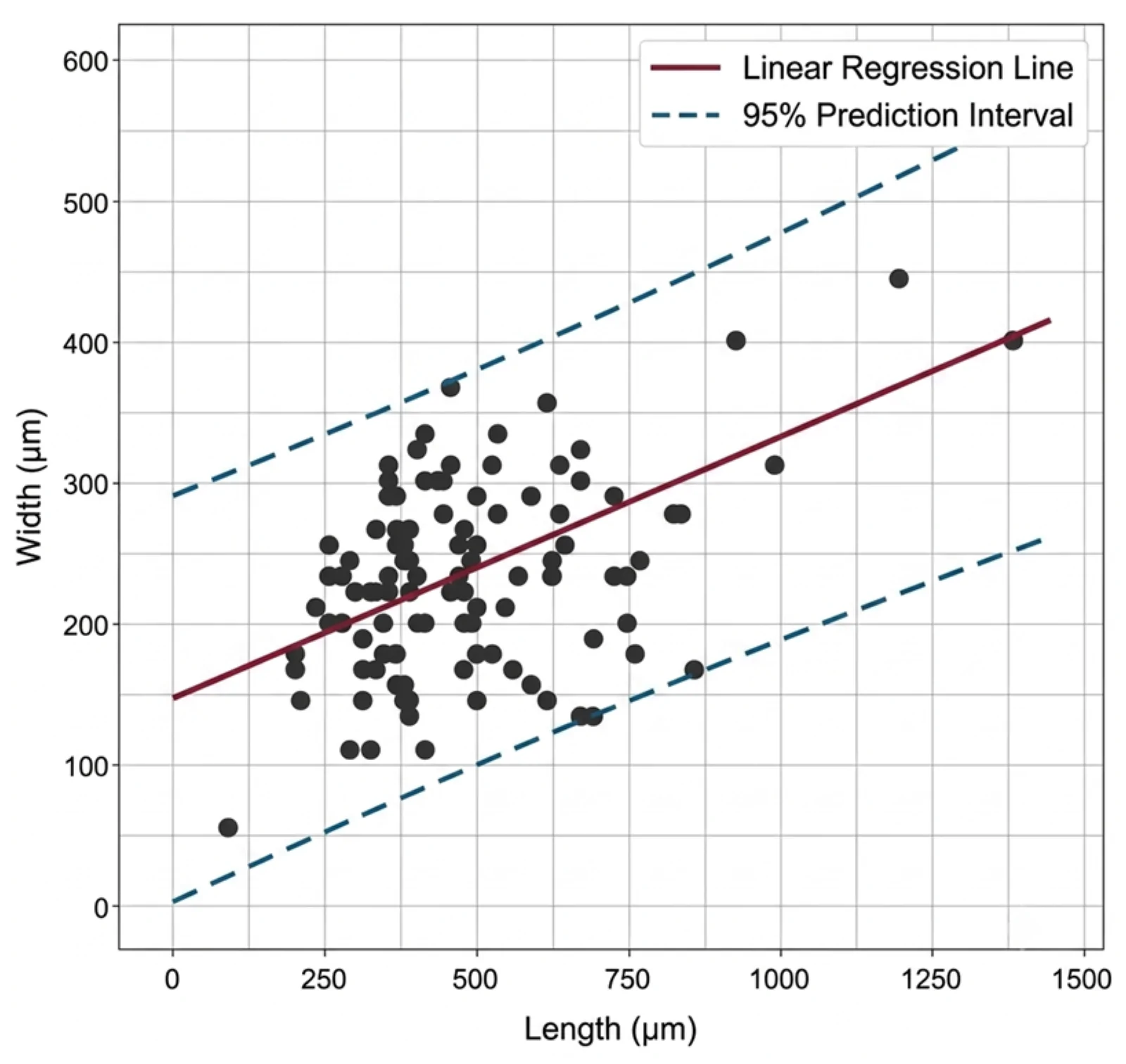
Regression for Width vs. Length.

Additionally, individual particle length and width measurements were obtained from SEM images, reporting at least 20 random particles from each clearance distance; then the information of three conditions was concentrated and is presented in Figure 5.

A positive linear relationship between particle length and width is observed in Figure 5, indicating that both dimensions increase proportionally throughout the evaluated operating conditions. The fitted regression suggests that larger particles tend to preserve similar geometric proportions, regardless of their absolute size. This relationship supports the hypothesis that the fragmentation process follows a common fracture pattern, generating particles with consistent morphological characteristics despite differences in clearance distance.

To further characterize particle morphology, additional information was obtained from the same particles observed in Figure 5 (see Table 2).

The average particle length increased from 424.1 µm for the 250 µm clearance distance to 499.7 µm and 880.2 µm for the 300 µm and 400 µm conditions, respectively. Similarly, average particle width increased from 203.1 µm to 260.2 µm and 386.4 µm. Despite these dimensional differences, the mean aspect ratio remained relatively constant, ranging from 2.02 to 2.37. These results indicate that increasing clearance distance increases particle dimensions while preserving the elongated morphology produced by the fragmentation process.

The morphological characterization obtained from SEM analysis is consistent with the laser diffraction results presented in Table 1. Both analyses demonstrate that increasing clearance distance generates progressively larger particles, while particle geometry remains relatively constant with the same shape. This agreement between direct image measurements and volumetric particle size distributions supports the conclusion that clearance distance is an effective parameter for controlling particle size generation without substantially modifying particle shape.

## 4. Discussion

The results demonstrate that during impact-based fragmentation, clearance distance is a crucial parameter for controlling dentin particle size. Laser diffraction measurements showed a progressive increase in $M_{\mathrm{volume}}$, $Dv_{10}$, $Dv_{50}$, and $Dv_{90}$ as clearance distance increased from 250 µm to 400 µm.

Particle size homogeneity was also strongly influenced by clearance distance. Based on the evaluated conditions, the 300 µm clearance produced the lowest span value (0.34), compared with 0.70 and 0.57 for the 250 µm and 400 µm conditions, respectively. Because span reflects the width of the particle size distribution, this result indicates that the 300 µm condition generated the most homogeneous particle population. Additionally, the volume distribution exhibited the narrowest peak under this condition, suggesting the optimal fragmentation efficiency, compared with the other conditions.

SEM analysis revealed elongated particle morphologies for all clearance distances. Quantitative measurements confirmed that both particle length and width increased with increasing clearance distance, consistent with the results observed by laser diffraction. However, the aspect ratio remained relatively constant, ranging from 2.02 to 2.37. These findings indicate that clearance distance primarily affects particle size rather than particle shape. The positive relationship between particle length and width further suggests that the fragmentation process follows a common fracture mechanism, producing geometrically similar particles across all operating conditions.

**Figure 6:**
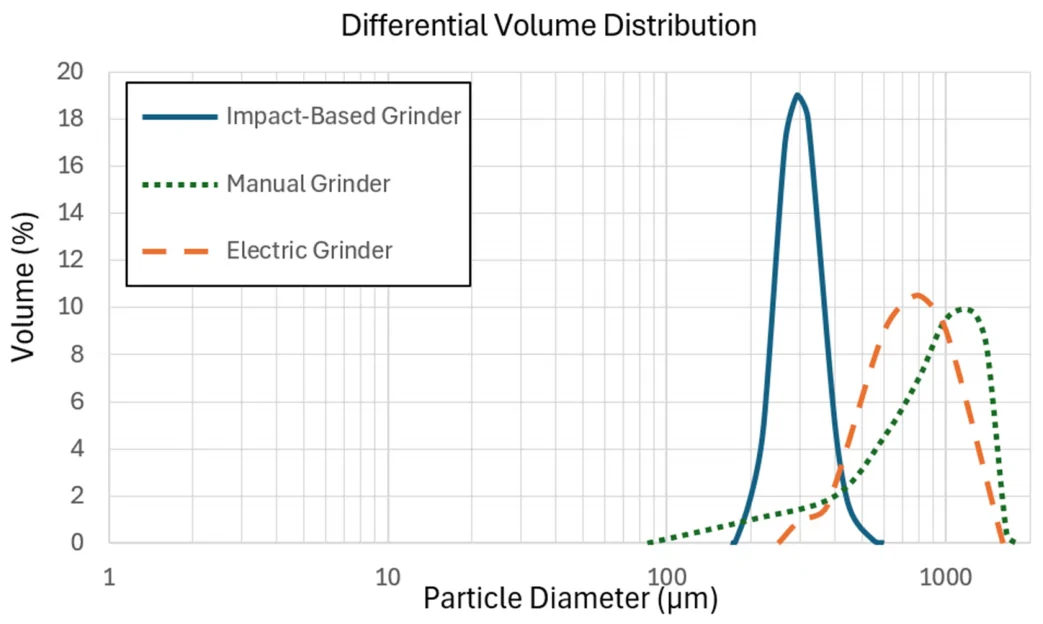
Grinder method comparison: (blue) Impact-Based Grinder; (green) Manual Grinder; and (orange) Electric Grinder [13].

Comparing the proposed impact-based grinder with other existing and proven grinding methods [13] also demonstrates some advantages (see Figure 6).

While manual and electric grinders produced broad particle size distributions, the impact-based system generated a narrower, concentrated distribution. This reduced variability indicates improved process control and greater reproducibility of the resulting particle population. In addition, the impact-based approach preserved particle morphology while allowing particle size to be adjusted through a well-defined geometric parameter (clearance distance).

Overall, the combined laser diffraction and SEM results demonstrate that controlled impact fragmentation provides an effective method for producing dentin particles with predictable dimensions and consistent morphology. Among the evaluated conditions, a clearance distance of 300 µm provided the most favorable balance between particle size and particle size homogeneity, supporting its use as the optimal operating condition for controlled dentin particle production.

The biological significance of controlling dentin particle size extends beyond manufacturing considerations, as particle dimensions directly influence the behavior of graft materials during bone regeneration. Previous studies have shown that particle size affects cellular migration, vascular penetration, resorption kinetics, and new bone formation. In general, smaller particles provide a greater specific surface area available for protein adsorption and cell attachment, while excessively large particles may reduce the biologically active surface available for remodeling processes [20,21,22,23,24,25]. Consequently, controlling particle size distribution is not only relevant from an engineering perspective but also from a regenerative standpoint.

The results obtained in the present study are particularly relevant because the 300 µm clearance condition generated particles with a mean diameter of approximately 290 µm and the most homogeneous particle population. This size is located near the lower boundary of the range commonly reported for dentin-derived grafting materials and bone substitutes intended for regenerative procedures [22,23,24,25]. Previous investigations have demonstrated that particle dimensions directly affect biological performance and bone healing outcomes. Leiblein et al. reported that graft granule size significantly influences bone regeneration behavior, while Kon et al. demonstrated that particle size affects bone augmentation potential and remodeling dynamics [20,21]. Therefore, the generation of particles concentrated around 300 µm may provide a favorable balance between surface availability and structural stability.

An important observation is that the 300 µm condition exhibited the lowest span value (0.34), indicating the narrowest particle size distribution among the evaluated conditions. Span is widely used as a descriptor of particle size distribution width and is calculated as (D90-D10)/D50 [31]. Lower span values indicate narrower distributions and therefore a more homogeneous particle population. Similar findings have been reported by Kruszelnicka et al., who observed that narrower distributions are associated with greater particle homogeneity during grinding processes [32]. Consequently, the low span value obtained in the present study suggests a superior degree of control over particle production.

The narrow peak observed in the volumetric distribution curve for the 300 µm condition may also indicate improved fragmentation efficiency. Studies from other particle processing fields have shown that systems characterized by narrow particle size distributions often exhibit more uniform behavior than systems with broader distributions, even when average particle size remains comparable [33]. Likewise, highly uniform particle populations are considered indicators of process consistency and optimized production conditions [34]. Although these observations originate from different technological applications, they support the relevance of obtaining narrow particle distributions when seeking biomaterial standardization.

Another aspect highlighted by the present results is the potential advantage of controlling particle generation directly during the fragmentation process rather than relying exclusively on post-processing sieving stages. According to the classification proposed by Minetti et al. [35], Smart Dentin Grinder and BonMaker belong to a group of systems that combine grinding and particle separation through sieve-based classification. In Smart Dentin Grinder, high-speed mechanical milling is followed by vibration-assisted sieving that retains particles outside the desired size range. Similarly, BonMaker employs crushing followed by manual particle separation through sieves of predetermined mesh sizes [35]. In contrast, the proposed impact-based system controls particle generation through a predefined mechanical clearance distance, allowing particle dimensions to be regulated during fragmentation itself.

The comparison with commercially available dentin-processing systems is also relevant from a clinical perspective. Dłucik et al. compared Smart Dentin Grinder, BonMaker, and Tooth Transformer and reported substantial differences in particle morphology and structural organization among the resulting biomaterials [28]. Smart Dentin Grinder and BonMaker predominantly rely on mechanical grinding and sieving processes, whereas Tooth Transformer combines mechanical treatment with chemical and thermal processing steps that alter the structure and degree of demineralization of the particles [28]. Despite these differences, all evaluated materials were considered clinically suitable for bone regeneration procedures [28]. Similarly, the present study demonstrated that impact-driven fragmentation produces particles with consistent elongated morphology while allowing for dimensional control through a single mechanical parameter.

The relevance of achieving controlled particle dimensions is further supported by the relationship between particle size and available surface area. Previous studies on bone substitute materials have shown that smaller particles provide greater surface availability for physicochemical interactions, ionic exchange, apatite formation, and cellular attachment [36]. In addition, Demir et al. reported that both particle size and particle size distribution influence microstructural homogeneity and material performance [36]. Therefore, the homogeneous particle population obtained under the 300 µm condition may represent a favorable characteristic for future regenerative applications.

From a manufacturing standpoint, reduced variability is commonly associated with improved quality and reproducibility. Zanobini et al. described variability as one of the principal factors affecting measurement quality and process consistency [37]. Likewise, Lamontagne and Fesnak emphasized that limiting variability is essential for obtaining reproducible products in advanced biomedical manufacturing systems [38]. Consequently, the reduced variability observed in the present impact-based fragmentation system may facilitate future standardization of dentin graft preparation protocols and contribute to more predictable material characteristics.

Finally, the clinical implications of controlled dentin particle production should be considered in the context of the intrinsic biological properties of dentin derived grafts. Autologous dentin exhibits osteoconductive and osteoinductive potential due to its collagen-rich matrix and the presence of growth factors involved in bone regeneration [14,15,16,17,18,19]. Mazzucchi et al. demonstrated that dentin particles release osteogenic molecules such as BMP-2, osteocalcin, and osteonectin, supporting their regenerative potential [27]. Therefore, combining these biological properties with improved granulometric control may contribute to the development of more predictable grafting materials. Although the present investigation focused on granulometric and morphological characterization, future studies should evaluate cellular response, bone formation, and osteointegration associated with the particle populations generated under the optimal 300 µm condition.

Although the present study demonstrates the capability of the proposed impact-based system to generate dentin particles with controlled granulometric characteristics, biological response, bone formation, and osseointegration were not evaluated. Therefore, future in vivo studies are required to determine the regenerative performance of the generated particles and to establish the relationship between particle size distribution and clinical outcomes.

## 5. Conclusions

This study demonstrated the feasibility of using a controlled impact-based fragmentation system for the production of dentin-derived particles intended for grafting applications. The results confirmed that clearance distance is an effective parameter for regulating particle size while maintaining consistent particle morphology.

Among the evaluated conditions, the 300 µm clearance distance provided the most homogeneous particle population and the best overall balance between particle size control and process consistency. These characteristics are particularly relevant for the standardization of dentin-derived biomaterials, where reproducible granulometric properties are desirable.

Compared with conventional grinding approaches, the proposed system offers an alternative strategy in which particle dimensions can be regulated directly through a controlled mechanical parameter. Therefore, controlled impact fragmentation represents a promising method for the production of dentin particles with predictable characteristics and potential application in future bone regeneration and grafting procedures.

Further studies should evaluate the biological performance, osteointegration potential, and regenerative response associated with the particle populations generated by this method.

## Figures and Tables

**Table 1:** Volumetric particle size distribution metrics of dentin particles obtained at different fragmentation clearance distances.

Parameter	Clearance Distance (250 µm)	Clearance Distance (300 µm)	Clearance Distance (400 µm)
$M_{volume}$	222 µm	289.6 µm	356 µm
${Dv}_{10}$	135.2 µm	244.9 µm	271.5 µm
${Dv}_{50}$	227.1 µm	285.8 µm	340.8 µm
${Dv}_{90}$	293.5 µm	340.8 µm	465.7 µm
Span	0.7	0.34	0.57

**Table 2:** Morphological characterization of fragmented dentin particles determined from SEM measurements under different clearance distances.

Clearance Distance	*n*	Mean Length L (µm)	Mean Width W (µm)	Mean Aspect Ratio (L/W)
250 µm	64	424.1 ± 144.6	203.1 ± 55.3	2.21 ± 0.92
300 µm	36	499.7 ± 154.7	260.2 ± 54.3	2.02 ± 0.88
400 µm	20	880.2 ± 291.0	386.4 ± 107.1	2.37 ± 0.81

## Data Availability

The original contributions presented in this study are included in the article. Further inquiries can be directed to the corresponding author.
